# LMDI Decomposition Analysis of E-Waste Generation in the ASEAN

**DOI:** 10.3390/ijerph182312863

**Published:** 2021-12-06

**Authors:** Gobong Choi, Taeyoon Kim, Minchul Kim

**Affiliations:** Green Technology Center, 17th Floor Namsan Square Bldg., 173, Toegye-ro, Jung-gu, Seoul 04554, Korea; gchoi0322@gtck.re.kr (G.C.); tykim@gtck.re.kr (T.K.)

**Keywords:** E-waste, waste management, environment policy, ASEAN, decomposition analysis

## Abstract

The economies of ASEAN member states are growing rapidly, and electrical and electronic waste (E-waste) generated from them are also showing a rapid increase. In this context, this study conducted an LMDI decomposition analysis on the amount of E-waste generated in ASEAN member countries from 2015 to 2019 and decomposed it into E-waste intensity, economic growth, and population effects. Then, based on analysis results, policy implications are suggested to improve their E-waste management. According to the analysis results, ASEAN countries can be classified into three groups. The first group includes Indonesia, the Philippines, Singapore, Thailand; economic growth was the main driving factor of E-waste increase in these countries. However, E-waste had also decreased due to the effect of E-waste intensity. The second group includes countries where economic growth was not the only driving factor for E-waste increase, but also where E-waste had increased due to the effect of E-waste intensity. These countries include Cambodia, Malaysia, and Viet Nam. Finally, the third group consists of countries where the effect of E-waste intensity is the main driving factor, including Brunei Darussalam, Lao PDR, and Myanmar. This research shows that ASEAN countries need policies that can effectively deal with the threat of E-waste as a result of high economic growth and policies that can improve intensity by reducing the generation of E-waste.

## 1. Introduction

As urbanization and industrialization proceed, and the level of income increases, more electrical and electronic equipment (EEE) is consumed, and the amount of electrical and electronic waste (E-waste) increases accordingly [1]. As Forti et al. [1] indicated, the amount of E-waste generation was 53.6 million metric tons (Mt) in 2019 globally, which had increased by 9.2 Mt since 2014 and is expected to reach 74.7 Mt in 2030. 

As indicated in the UNEP Basel Convention [2], although hazardous substances contained in EEE have been reduced due to the national legislation in many countries, hazardous substances such as lead, cadmium, and mercury may still exist in E-waste. E-waste is classified as hazardous waste [2], and it affects both the environment and human beings negatively if handled and treated inappropriately [3,4]. Thus, E-waste properly through relevant laws and policies to minimize or avoid the harmful effects. Indeed, some developed countries have established and enforced Acts, such as the EU Directive on Waste Electrical and Electronic Equipment (Directive 2012/19/EU). Japan and the Republic of Korea have also legislated such laws. In Japan, the Act on Promotion of Recycling of Small Waste Electrical and Electronic Equipment (Act No. 57 of 2012) has been enforced, and Korea has the Act on Resources Circulation of Electrical and Electronic Equipment and Vehicles (Act No. 15842, 16 Oct. 2018) in force. Moreover, E-waste has been managed internationally. It is included in the hazardous waste list A in the Annex VIII of the Basel Convention, an international convention that prevents transboundary movements of hazardous waste. Furthermore, reducing the generation of E-waste is an international goal since it is included and tracked as a sub-indicator of Target 12.4 of the United Nation’s Sustainable Development Goals (SDGs).

In terms of general waste hierarchy, including E-waste, the priority of waste management is to prevent and reduce the amount of waste [5,6]. This concept of priority has been established in the guidelines or plans of many countries. The EU Waste Framework Directive (Directive 2008/98/EC) specifies that “prevention” has the highest priority in Article 4 [7] (p. 6), and the 1st Basic Plan on Resource Circulation in Korea also sets “reducing the generation of waste fundamentally” as the top priority of the policy [8] (p. 40). 

However, it may not be easy to fundamentally reduce E-waste depending on the factor contributing to E-waste generation. For example, if the inefficient use of EEE in the industrial sectors is the main factor, a country can promote efficiency, thereby reducing the amount of E-waste generated with the same yields. On the contrary, if economic growth or population growth is the main factor, restraining future growth could reduce E-waste generation. However, this is not a realistic waste management policy. Instead, promoting the efficient use of EEE, recycling, or enforcing further treatment methods to avoid exposure to hazardous components of E-waste can be more reasonable.

Thus, to resolve the E-waste problem in a country, it is necessary to identify the factors that contribute to the increase of E-waste and to develop, modify, and supplement waste management policies accordingly. To identify these factors, decomposition analysis serves as one of the most widely used methodologies as it utilizes various factors, including economic development, to look at the increases or decreases in a targeted subject [9,10,11]. Decomposition analysis has been applied to find out the driving factors of carbon emissions which is also considered as one of the environmentally undesirable outputs (González et al. [12]; Ma [13]; Mairet and Decellas [14]; Rogan et al. [15]; Quan et al. [16]). However, limited decomposition studies have been conducted on waste generation, including E-waste generation. Brix and Bentzen [17] conducted an index decomposition analysis (IDA) to figure out the factors that affect the amount of waste generated in Denmark, and He et al. [18] performed a structural decomposition analysis (SDA) on the waste generation in Australia. Regarding E-waste generation, it is hard to find research that focuses on decomposition analysis. Only some quantitative analyses have been conducted, such as Boubellouta and Kusch-Brandt’s study [19], which tried to identify variables affecting the generation of E-waste through an econometric analysis, and Kusch and Hills’ research [20], which studied the relationship between E-waste and gross domestic products.

The purpose of this study is to find out what factors caused the increase or decrease in E-waste generation and to provide policy implications for E-waste management. In order to achieve this purpose, this study conducted the LMDI (Logarithmic Mean Divisia Index) decomposition analysis on E-waste generation from 2015 to 2019 and decomposed the increases or decreases in E-waste generation into three factors: E-waste intensity, economic growth, and population.

In particular, this study focused on the ASEAN countries, as the ASEAN countries are rapidly growing developing countries. As Park et al. [21] indicated, E-waste generation in developing countries is increasing rapidly. However, developing countries have relatively insufficient waste management systems compared to developed countries, so continuous legislation and system improvement for E-waste management are needed. Therefore, this study intends to provide policy implications for E-waste management in ASEAN countries and at the same time contribute academically as a quantitative study on E-waste. In this process, although Singapore is a high-income country, as it is a member state of ASEAN, has been included in the study.

This paper is organized as follows. In Section 2, the trend of E-waste generation in ASEAN countries is briefly explained. Section 3 describes the LMDI decomposition methodology and the data used in the analysis. Section 4 explains and discusses the results of the LMDI decomposition, and Section 5 concludes this study with a summary.

## 2. The E-Waste Generation in ASEAN

According to the Country Sheets in The Global E-waste Statistics Partnership [22] website, as shown in Table 1, the worldwide amount of E-waste generated in 2015 was 46,353 thousand tons, and it increased by 15.6% and reached 53,601 thousand tons in 2019. During the same period, the amount of E-waste generated in the United States increased by 6.4%, and that of Europe increased by 5.4%, showing a lower increase rate than the global rate of increase. On the other hand, the amount of E-waste generated in ASEAN, which consists of 10 Southeast Asian countries, increased by 19.8%, showing a 4.2%p higher growth rate than the world’s average annual growth rate.

**Figure 1:**
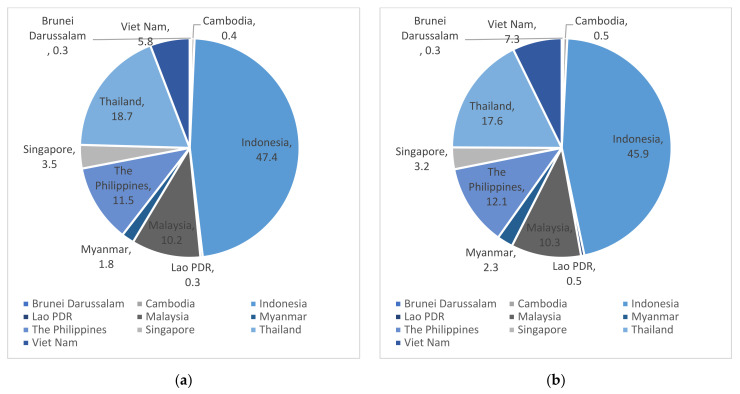
Shares of the E-waste generation amounts in ASEAN: (**a**) the shares in 2015; (**b**) the shares in 2019.

From 2015 to 2019, the total amount of E-waste generated in ASEAN increased sharply, but a sharper increase was confirmed for some member states. The E-waste in ASEAN as a whole increased by 19.8% from 2015 to 2019, but Cambodia (46.2%), Lao PDR (70.0%), Malaysia (21.3%), Myanmar (51.9%), the Philippines (25.4%), and Viet Nam (49.4%) showed a steeper increase, as shown in Table 1. On the other hand, during the same period, Brunei Darussalam (12.5%), Indonesia (16.1%), Singapore (10.8%), and Thailand (12.9%) showed a relatively lower increase compared to other ASEAN countries. These countries, except for Indonesia, showed lower increase rates compared to the world average, but still being higher than the United States and Europe. Accordingly, as shown in Figure 1, the share of E-waste generation of Cambodia, Lao PDR, Malaysia, Myanmar, the Philippines, and Viet Nam increased slightly, and the share of the rest decreased. Indonesia, which generates the most E-waste in ASEAN, has a rate of increase of 16.1%, which is lower than the overall increase rate of ASEAN, so its share diminished to 45.88%, but it still occupies almost half of E-waste generation in ASEAN.

## 3. Methodology and Data

### 3.1. LMDI Decomposition

In this study, the amount of E-waste generation in ASEAN countries was decomposed by using the Logarithmic Mean Divisia Index (LMDI) decomposition method. LMDI decomposition analysis is a methodology used in many ways to decompose and measure the impact of human activities on the environment, such as energy consumption and carbon emissions, by specific factors that affect increases or decreases in them. Among index decomposition analyses, as Ang [23] indicated, LMDI decomposition has the advantage that residuals do not remain after analysis, which means perfect decomposition. Thus, by applying the LMDI decomposition method, the amount of E-waste generation can be fully described by the factors considered.

In order to conduct the LMDI decomposition analysis, an identity equation composed of the object to be analyzed and its factors should be constructed. Many environments and energy studies have set the identity equation based on the “Impact = PAT” identity equation (Equation (1)), which was introduced by Ehrlich and Holden [24,25]

(1) Impact=P×A×T 

This identity equation sets environmental impact as the object and three human activities (population, P; affluence, A; technology, T) as three basic factors. This study also used the basic identity equation. The amount of E-waste generated can be considered as the environmental impact, and P and A can be set as population and affluence, respectively, likewise the factors in the basic identity equation. As several studies include carbon intensity or energy intensity of the economy as technology, technology (T) can be replaced by the E-waste generation relative to the size of the economy, which means E-waste intensity of the economy is similar to the indicator “waste intensive economy” in Bastos et al. [26] (p. 23). Then, the identity equation in this study can be written as Equation (2)

(2) $$EEW=POP\times \frac{GDP}{POP}\times \frac{EEW}{GDP}=P\times G\times I\;$$

where EEW, POP, and GDP stand for E-waste generation, population, and gross domestic product, respectively. In this study, the affluence is defined as GDP per capita (GDP/POP), and E-waste intensity is defined as E-waste generation per GDP (EEW/GDP).

According to Equation (2), the amount of E-waste generated in a country can be explained by population, economic growth, and E-waste intensity. As the population increases, the total amount of EEE consumed in a country increases, thus, increasing their wastes. In addition, as a country’s income level rises, EEEs that may have the characteristics of normal goods are consumed more, and the amount of E-waste increases as well. Finally, the increases in the E-waste intensity of a country mean deterioration of efficiency, so the country generates more E-waste relative to its economic size.

When conducting the LMDI decomposition analysis, using data of E-waste generation by sectors or by EEE (e.g., specific E-waste streams), the E-waste generation can be decomposed, including economy “structure” factor or E-waste “composition” factor. However, E-waste data used in this study is available at the aggregated level, so including additional factors that can explain the E-waste generation is quite limited. This is a limitation of this study, and if there are detailed data on E-waste, further research considering additional factors can be carried out, and more diverse policy implications can be provided.

The LMDI decomposition is divided into additive decomposition and multiplicative decomposition. The additive decomposition shows an absolute amount that each factor contributes to increases or decreases in E-waste generation between the base year (t = 0) and the comparison year (t = T). Thus, the sum of each factor’s effects between two years equals the total absolute amounts of changes in E-waste generation during the same period. The multiplicative decomposition shows an increase or decrease ratio caused by each factor between the years. Thus, the total product of each factor’s ratio between two years equals the total ratio of changes in E-waste generation during the same period. Multiplicative decomposition does not show the absolute amount of changes, but it is useful when comparing two or more countries since it controls the scale of a country and only shows the multiplicative changes based on the reference year. This study conducted the LMDI decomposition analysis both additively and multiplicatively.

In order to derive the additive form of LMDI decomposition from Equation (2), the result of the total differentiation of Equation (2) is written as Equation (3). After modifying Equation (3) into the form of differentiated logarithmic function (Equation (4)) and then integrating from the base year (t = 0) to the comparison year (t = T) (Equation (5)), the final additive decomposition equation can be derived (Equation (6)). As shown in Equation (6), the total change of EEW (ΔEEW) expressed as the difference between the amount of E-waste generated is decomposed into the changes caused by the intensity effect (ΔEEWI), growth effect (ΔEEWG), and population effect (ΔEEWP) at the same period and their sum are equal to the total change. v* in Equation (6) is the logarithmic mean weight used when transforming Equation (5) into Equation (6) by applying the mean value theorem of integrals.

(3) dEEW=PG×dI+PI×dG+GI×dP 

(4) $$dEEW=EEW\frac{dI}{I}+EEW\frac{dG}{G}+EEW\frac{dP}{P}=EEW\times d\ln I+EEW\times d\ln G+EEW\times d\ln P\;$$

(5) ∫dEEW=∫EEW×dlnI+∫EEW×dlnG+∫EEW×dlnP 

(6) $$\Delta EEW=EEW(T)-EEW(0)=v^{*}\ln\frac{I(T)}{I(0)}+v^{*}\ln\frac{G(T)}{G(0)}+v^{*}\ln\frac{P(T)}{P(0)}=\Delta EEW_{I}+\Delta EEW_{G}+\Delta EEW_{P}\;\;\mathrm{where}v^{*}=EEW^{*}=\frac{EEW(T)-EEW(0)}{\ln EEW(T)-\ln EEW(0)}\;$$

On the other hand, deriving the multiplicative form of LMDI decomposition from Equation (2) is as follows. After modifying Equation (2) into the form of differentiated logarithmic function (Equation (4)) and dividing both sides by EEW, Equation (7) is derived. When integrating both sides of Equation (7) from the base year (t = 0) to the comparison year (t = T) (Equation (8)), the equation can be written as Equation (9). Applying exponential function on both sides of Equation (9), then the final multiplicative decomposition equation can be derived (Equation (10)). As shown in Equation (10), the total change ratio of EEW (ΔEEW) is decomposed into the changes caused by the three effects, and their total product is equal to the total change. Here, the logarithmic mean weight is not required or can be thought of as 1. This is because this study does not consider economic sectors or specific waste streams due to the lack of data, so the derivation gets simplified. Therefore, when considering the sectors or streams, the total amount of E-waste generation is expressed as the summation of each sector or stream, the multiplicative form, and of course, the additive form of LMDI decomposition with the weight should be re-derived.

(7) $$\frac{dEEW}{EEW}=d\ln EEW=d\ln I+d\ln G+d\ln P\;$$

(8) ∫dlnEEW=∫dlnI+∫dlnG+∫dlnP 

(9) $$\ln\frac{EEW(T)}{EEW(0)}=\ln\frac{I(T)}{I(0)}+\ln\frac{G(T)}{G(0)}+\ln\frac{P(T)}{P(0)}\;$$

(10) $$\Delta EEW=\frac{EEW(T)}{EEW(0)}=\frac{I(T)}{I(0)}\frac{G(T)}{G(0)}\frac{P(T)}{P(0)}=\Delta EEW_{I}\times \Delta EEW_{G}\times \Delta EEW_{P}\;$$

### 3.2. Data

All data used in this study were collected for 10 ASEAN countries (Brunei Darussalam, Cambodia, Indonesia, Lao PDR, Malaysia, Myanmar, the Philippines, Singapore, Thailand, and Viet Nam), which are the geographic scope of this study. E-waste generation, GDP, and population data are required to analyze according to the presented methodology in Section 3.1.

The data on electronic waste generated from 2015 to 2019 provided on the GESP website [22], Forti et al. [1] and Baldé et al. [27]’s Global E-waste Monitor is used for the amount of E-waste generated. The GESP website discloses the estimated statistics on the amount of E-waste generated by each country and continent from 2015 to 2019 as a Country Sheet. According to Forti et al. [1] and Baldé et al. [27], the data on electronic waste generated is an estimated statistic, calculated by the amount of electrical and electronic equipment produced, exported, and imported in a country and the lifetime distribution of each product. Thus, there is expected to be a difference in quality from the data constructed by measured statistics or survey statistics in a country. Despite this fact, there is no such data publicly available, so this study has academic significance in that it was analyzed based on the best available data in a situation where national statistical data is non-existent.

Data from World Bank [28] are used for Gross Domestic Product (GDP) and population data for each country in ASEAN from 2015 to 2019. For GDP, constant US dollar data with inflation-adjusted as of 2010 was used, and total population data was used for population.

## 4. Decomposition Results

### 4.1. Additive Decomposition

**Figure 2:**
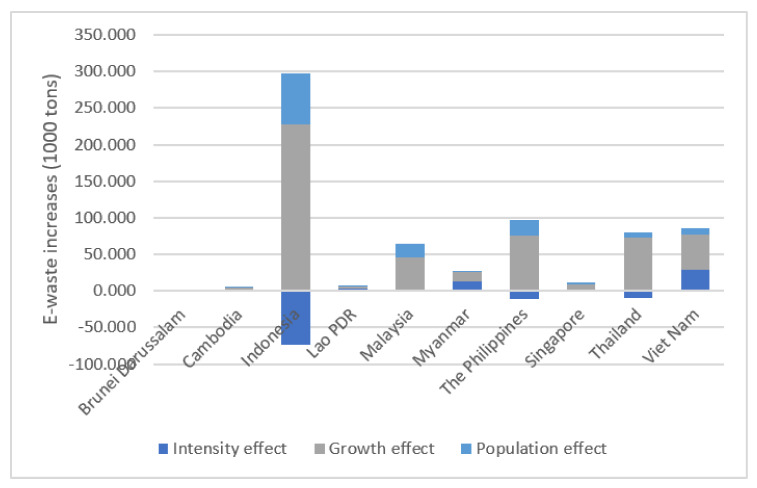
E-waste increases by effect (Additive, 2015–2019).

**Figure A1:**
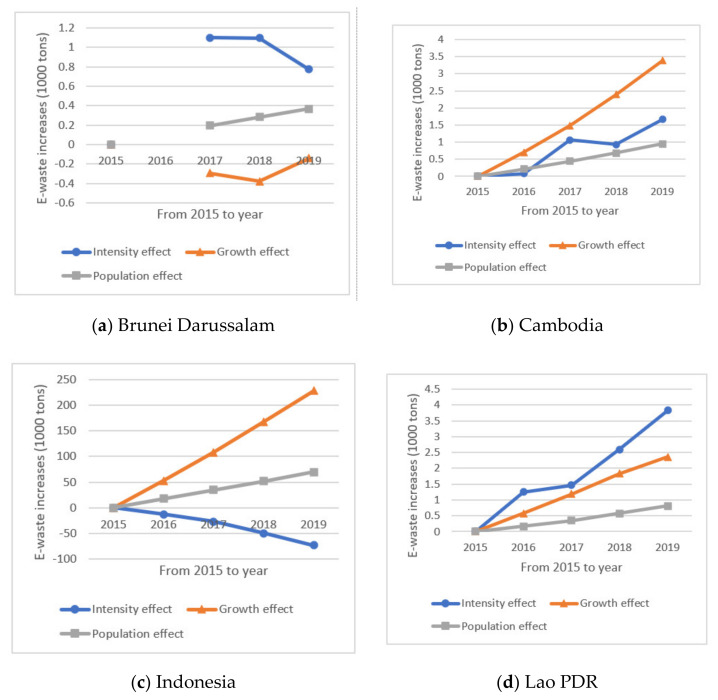

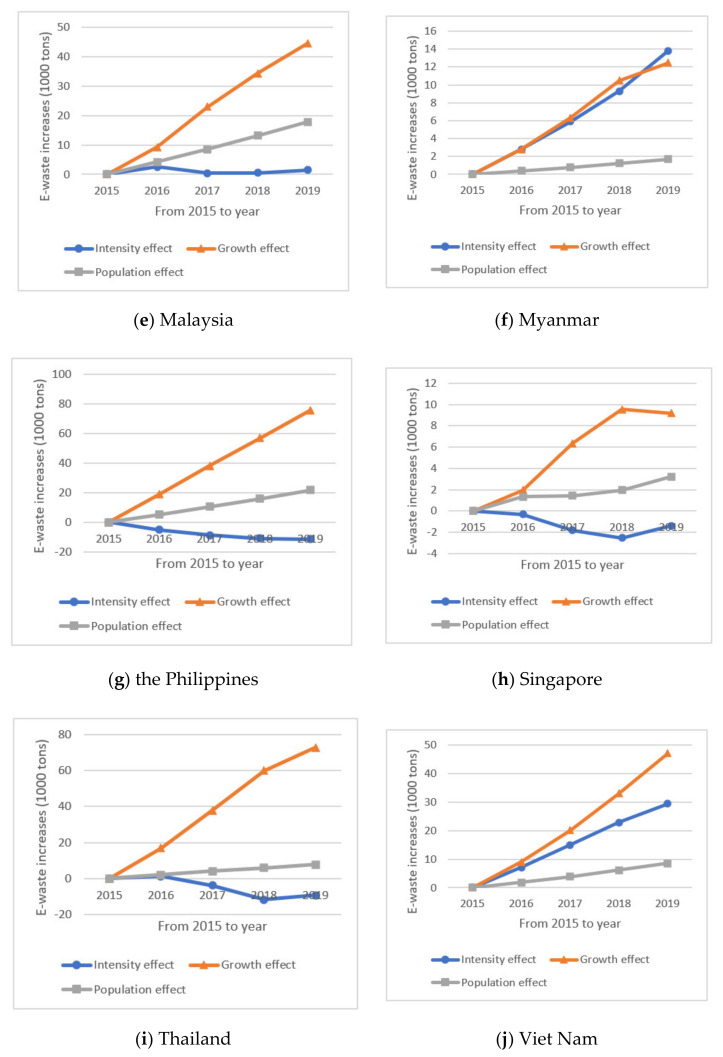
The results of additive LMDI decomposition analysis from 2015 to each comparison year: (**a**) Brunei Darussalam; (**b**) Cambodia; (**c**) Indonesia; (**d**) Lao PDR; (**e**) Malaysia; (**f**) Myanmar; (**g**) the Philippines; (**h**) Singapore; (**i**) Thailand; (**j**) Viet Nam.

Table 2 and Figure 2 show the results of additive LMDI decomposition analysis for the E-waste generation in ASEAN. The results show the E-waste increase between 2015 and 2019, and the results between each comparison year from the same base year (2015) are presented in Figure A1, Appendix A. The country with the largest increase in the amount of E-waste generated from 2015 to 2019 was Indonesia, followed by the Philippines and Viet Nam. The E-waste in Indonesia increased by a total of 224 thousand tons during the same period, while that in the Philippines and Viet Nam increased by 86 and 85 thousand tons, respectively. The country with the least increase in E-waste was Brunei Darussalam, increasing by 1 thousand tons.

Investigating the increase in E-waste by factors, the countries with the most significant increase due to economic growth were Indonesia (228.0 thousand tons), and the Philippines (75.7 thousand tons), and Thailand (72.7 thousand tons) followed. In the case of Brunei Darussalam, about 0.1 thousand tons of E-waste decreased due to economic growth. Brunei Darussalam had recorded negative growth until 2016 due to a sharp drop in oil prices in 2014. It had achieved economic recovery, recording higher GDP in 2019 than in 2015, but GDP per capita has not yet recovered to the level of 2015. The effect of the population appeared in the order of Indonesia, the Philippines, and Malaysia. During the same period, Indonesia’s E-waste increased by 69.6 thousand tons due to the population effect, while the Philippines and Malaysia’s E-waste increased by 21.7 and 17.9 thousand tons, respectively. For the effect of E-waste intensity, Viet Nam showed the most considerable amount of E-waste generated by intensity, and their E-waste increased by 29.5 thousand tons. In terms of the E-waste intensity, after Viet Nam, Myanmar (13.8 thousand tons), and Lao PDR (3.8 thousand tons) followed. In some countries, the amount of E-waste generation decreased due to the intensity effect, and the amount of reduction (73.6 thousand tons) was the largest in Indonesia.

### 4.2. Multiplicative Decomposition

**Figure 3:**
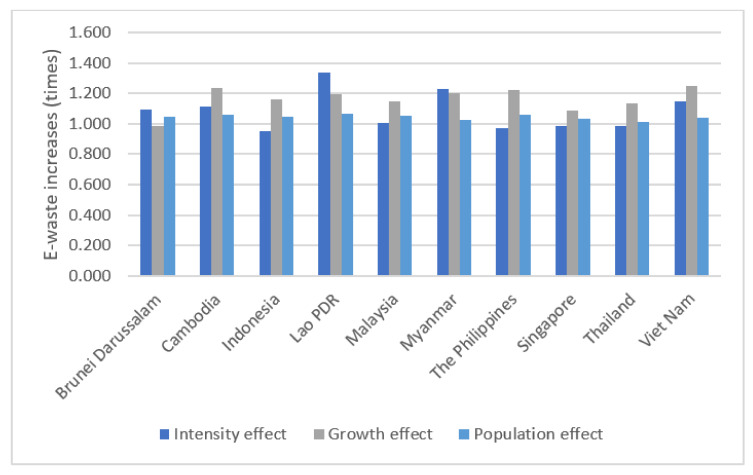
E-waste increases by effect (Multiplicative, 2015–2019).

When the amount of E-waste generated is decomposed in a multiplicative way, the increase or decrease of the comparative year compared to the base year is expressed as a ratio. Thus, the effect of various national scales can be appropriately controlled. The multiplicative LMDI decomposition analysis results between 2015 and 2019 are shown in Table 3 and Figure 3. The country with the most significant increase in E-waste in 2019 compared to 2015 was Lao PDR, a total increase of 1.7 times. After Lao PDR, the E-waste in Myanmar and Viet Nam increased by 1.519 and 1.494 times, respectively. On the other hand, the E-waste in Indonesia, where the most significant amount of E-waste was generated in additive decomposition, increased only by 1.161 times during the same period.

Investigating the increase in E-waste by factors, the effect of economic growth was the largest in Viet Nam, and it increased by 1.249 times in 2019 compared to 2015. After Viet Nam, the E-waste in Cambodia and the Philippines increased by 1.238 times and 1.220 times due to economic growth. There was no significant difference in the effect by population among ASEAN countries. The country with the most considerable population effect was Lao PDR, where the amount of E-waste due to the population effect increased by 1.064 times. The country with the smallest population effect was Thailand, where their E-waste increased by 1.013 times. The intensity effect was greatest in Lao PDR, and the amount of E-waste increased by 1.336 times from 2015 to 2019. Next, the E-waste by intensity effect increased by 1.229 times in Myanmar and 1.149 times in Viet Nam. The country with the lowest intensity effect was Indonesia, where the amount of E-waste due to the intensity effect increased by 0.952 times. In other words, in Indonesia, the amount of E-waste decreased the most in both absolute and ratio due to the intensity effect. It is judged that the explosive increase of national regulations regarding hazardous waste management during the period of 2008–2009, such as Act No. 18/2008 Waste Management and Ministry of Environment Regulation No. 30/2009 concerning Implementation of Permit System and Monitoring of Hazardous Waste, is positively attributed to the reduction of intensity effect regarding the E-waste situation in Indonesia.

### 4.3. Classification by the Main Driving Factor

Based on the factors that had the most significant impact from 2015 to 2019, ASEAN countries can be divided into two groups. The first group is the country where the intensity of E-waste contributed the most to the increase in E-waste generation, which is three countries: Brunei Darussalam, Lao PDR, and Myanmar. Economic growth was the second driving factor among the countries belonging to the first group, excluding Brunei Darussalam. Brunei Darussalam is the only country where the amount of E-waste had decreased due to the effect of economic growth. During the same period, the gross economic level increased, but GDP per capita decreased slightly, resulting in a negative economic growth effect on the E-waste generation.

The second group is the country where economic growth contributed the most to the increase in the E-waste generation, and the remaining seven countries (Cambodia, Indonesia, Malaysia, the Philippines, Singapore, Thailand, and Viet Nam) are included. In the second group, except for Cambodia and Viet Nam, the effect of E-waste intensity was the lowest effect among the three effects, and in Cambodia and Viet Nam, the effect of intensity was the second driving factor.

### 4.4. Classification by the E-Waste Intensity

Among the factors that explain the increase in E-waste generation, a factor close to a practical policy variable in terms of waste management is the E-waste intensity. The increase in E-waste due to the intensity effect means that the amount of E-waste generated increases relative to the national economy level. Therefore, there is room for improving the intensity through good policies that promote efficient input and use of electrical and electronic products and reduce the E-waste generation. On the other hand, economic growth and population are difficult to suppress relatively and practically through policies.

Based on the effect of E-waste intensity close to policy variables, ASEAN countries can be divided into two groups. The first group is the countries where the generation of E-waste had decreased due to the intensity effect, and Indonesia, the Philippines, Singapore, and Thailand fall into this group. The second group is the countries where the amount of E-waste had increased due to the intensity effect, and Brunei Darussalam, Cambodia, Lao PDR, Malaysia, Myanmar, and Viet Nam belong to this group.

### 4.5. Discussion Based on the Classification Results

When country classification by main driving factor and E-waste intensity are combined, ASEAN member states can be divided into three groups.

Countries included in the first group, Indonesia, the Philippines, Singapore, and Thailand, are countries where economic growth is the main driving factor of E-waste increases, but E-waste had decreased due to the effect of E-waste intensity. E-waste in the first group had been increasing due to the economy’s expansion despite the improvement in E-waste intensity. Thus, E-waste management policies need to be strengthened to treat the E-waste generated effectively and reduce the amount of E-waste going to the final disposal. Introducing advanced technology through international technology transfer and expanding the hazardous waste treatment facilities operated by the local or central government can be a good example. Further, subsidizing the recycle and recovery sector of E-waste to settle down and invigorate them can be considered.

However, to promote recycling and recovery, it is a priority to prepare a systematic E-waste collection system. The E-waste collection services in Indonesia, the Philippines, and Thailand are mainly performed by the informal sector [29] (pp. 166, 195, 202), making waste management in a country difficult at the government level. However, as there is a high dependence on the informal sector, enforcement of policies such as business bans without appropriate alternative services may bring about social chaos. In addition, it may be difficult in terms of money and human resources to prepare a formal sector to replace them in a short period. 

Instead, a policy issuing licenses to appropriate informal sectors can be considered. Further, providing appropriate education and support for waste collection and treatment can be good options. By doing so, the informal sector can conduct business in the formal system while preventing harmful effects to public health. At the same time, it is necessary to supervise and monitor the licensed (informal) sector to prevent inadequate behaviors. It also needs to make the licensed (informal) sector report the information of E-waste, such as amount and items, to manage the whole waste management system.

The countries belonging to the first group also need to reduce their E-waste intensity continuously. Among these countries, except Singapore (0.34), the intensity of E-waste exceeds 1, and their intensities are still higher than that of high-income countries such as Korea (0.55) and Japan (0.41), as shown in Table 4. Therefore, Indonesia, the Philippines, and Thailand need to prepare and steadily implement policies to reduce the intensity of E-waste to maintain the current trend. For instance, in the Philippines, the Final Draft Guideline on the Environmentally Sound Management (ESM) of Waste Electrical and Electronic Equipment (WEEE) has been formulated [1] (p. 72). This guideline needs to be passed and implemented shortly to reduce the amount of E-waste, as established in the final draft as an objective.

The second group includes countries where economic growth is the main driving factor for E-waste increases, and at the same time, E-waste increased due to the effect of E-waste intensity. Countries that fall into the second group are Cambodia, Malaysia, and Viet Nam. Countries belonging to the second group need to strengthen their policies to reduce the final disposal of E-waste, and it is also necessary to prepare a systematic E-waste collection system. For example, in Cambodia, the enforcement is ineffective due to the lack of awareness and knowledge of the laws [29] (p. 185), so implementing their latest E-waste management law, Sub-decree on Electrical and Electronic Equipment Waste Management, could be difficult. Thus, providing a capacity-building program can be one method to help the officials and the firms who serve E-waste management to raise their competency for understanding and implementing the law.

In addition, the countries included in the second group need to introduce policies that can improve E-waste intensity more strongly than in the first group. The countries in the second group, except Malaysia, tend to increase in the effect of E-waste intensity continuously. Therefore, it is necessary to check overall economic activity, such as how many electrical and electronic products have been purchased, how much life span, and how many product repair services are used. In addition, it may also be a good idea to benchmark the related policies or management systems of the countries in the first group. In the case of Malaysia, the E-waste intensity effect was showing at a level close to 0, and it is judged that Malaysia is at a crossroads where E-waste can be reduced by improving the intensity. As Honda et al. [29] pointed out, in Malaysia, the electrical and electronics industry is a key manufacturing sector that contributes to their economy. Therefore, extending repair services for home appliances or electronic devices together with the companies in the industry and ensuring access to repair services can be a proper way to reduce the E-waste intensity effect.

The third group includes countries where E-waste intensity is the main driving factor. Countries belonging to the third group are Brunei Darussalam, Lao PDR, and Myanmar. These countries can be interpreted as countries in which E-waste intensity had become worse at a higher level than the improvement of the economic level. However, as Brunei Darussalam suffered a recent recession and Myanmar experienced a slowdown in economic growth in 2019, Brunei Darussalam and Myanmar may have an exceptional result in the third group. However, in Myanmar, as Forti et al. [1] stated, since there is no regulation on E-waste and E-waste is not classified as hazardous waste, it is necessary to prepare a regulation on E-waste as soon as possible. In the case of Lao PDR, no policies or regulations support the 3R (Reduce, Reuse, Recycle) principles or approaches to waste-to-resources at the government or city level [30] (p. 5). If the effects of economic growth and E-waste intensity are continuously maintained, the amount of E-waste is highly likely to increase significantly. Thus, the laws and policies related to E-waste should be prepared shortly from ex-ante management rather than the aspect of ex-post management in Lao PDR.

## 5. Conclusions

In this study, the LMDI decomposition analysis was performed on E-waste generation in ASEAN countries from 2015 to 2019, and the increase in E-waste generation was decomposed into three factors: intensity, economic growth, and population.

Analysis results show that E-waste generation increased significantly in ASEAN countries due to economic growth and the E-waste intensity effect rather than the population effect. In particular, from 2015 to 2019, Cambodia, Indonesia, Malaysia, the Philippines, Singapore, Thailand, and Viet Nam had the most significant economic growth effect among the three factors; Brunei Darussalam, Lao PDR, and Myanmar had the most enormous E-waste intensity effect. When comparing ASEAN countries, in terms of quantity, Indonesia had the most significant increase of E-waste due to the economic growth effect and population effect, while Viet Nam had the largest increase of E-waste due to the intensity effect. However, when comparing ASEAN countries based on the growth rate through multiplicative decomposition, Viet Nam had the largest economic growth effect, and Lao PDR had the most considerable intensity and population effect.

In particular, as a result of this decomposition analysis, the ASEAN countries could be divided into three groups. The first group includes Indonesia, the Philippines, Singapore, and Thailand, where economic growth was the main driving factor of E-waste increases, but E-waste had decreased due to the E-waste intensity effect. Unlike the first group, the second group consists of countries in which E-waste had increased due to the E-waste intensity effect, including Cambodia, Malaysia, and Viet Nam. The third group includes the countries where the effect of E-waste intensity is the main driving factor: Brunei Darussalam, Lao PDR, and Myanmar. Therefore, a fundamental E-waste reduction policy is needed to improve the E-waste intensity as a whole in ASEAN countries despite the decrease of the E-waste intensity of the first group. Moreover, the provision of a treatment system is also necessary.

In addition, the member states of ASEAN countries need efforts to establish and improve a waste monitoring system that includes E-waste. ASEAN countries lack monitoring systems essential to overall waste management, leading to difficulty in obtaining reliable E-waste tracking data for each country. This study used estimated data, which could be different from the actual E-waste generation in ASEAN countries. If the waste monitoring system is built and improved in each country through close cooperation between ASEAN states, publicly available and easily accessible data can be built in response to the Questionnaire on Environment Statistics of UNSD and UNEP. Moreover, this will enable more qualitative research on ASEAN countries that could lead to more specific policy implications on waste management and public health.

## Figures and Tables

**Table 1:** Changes in the E-waste generation.

Country	E-Waste Generation (1000 Tons)		Total Growth Rate (2015–2019, %)	Compound Annual Growth Rate (%)
	2015	2019		
Brunei Darussalam	8	9	12.5	3.0
Cambodia	13	19	46.2	10.0
Indonesia	1394	1618	16.1	3.8
Lao PDR	10	17	70.0	14.2
Malaysia	300	364	21.3	5.0
Myanmar	54	82	51.9	11.0
The Philippines	339	425	25.4	5.8
Singapore	102	113	10.8	2.6
Thailand	550	621	12.9	3.1
Viet Nam	172	257	49.4	10.6
ASEAN	2942	3525	19.8	4.6
Europe	11,393	12,013	5.4	1.3
United States	6502	6918	6.4	1.6
World ^1^	46,353	53,601	15.6	3.7

^1^ It is calculated by the sum of the E-waste generation in the 6 continents, Africa, Asia, Europe, Americas, and Oceania. Source: The Global E-waste Statistics Partnership [22].

**Table 2:** LMDI decomposition result: Additive decomposition from 2015 to 2019.

Country	Total Change (Thousand Tons)	Additive Decomposition (Thousand Tons)		
		Intensity Effect	Growth Effect	Population Effect
Brunei Darussalam	8	9	12.5	3.0
Cambodia	13	19	46.2	10.0
Indonesia	1394	1618	16.1	3.8
Lao PDR	10	17	70.0	14.2
Malaysia	300	364	21.3	5.0
Myanmar	54	82	51.9	11.0
The Philippines	339	425	25.4	5.8
Singapore	102	113	10.8	2.6
Thailand	550	621	12.9	3.1
Viet Nam	172	257	49.4	10.6

**Table 3:** LMDI decomposition result: Multiplicative decomposition from 2015 to 2019.

Country	Total Change (Times)	Multiplicative Decomposition (Times)		
		Intensity Effect	Growth Effect	Population Effect
Brunei Darussalam	1.125	1.095	0.983	1.044
Cambodia	1.462	1.111	1.238	1.062
Indonesia	1.161	0.952	1.164	1.047
Lao PDR	1.700	1.336	1.196	1.064
Malaysia	1.213	1.005	1.144	1.055
Myanmar	1.519	1.229	1.205	1.026
The Philippines	1.254	0.970	1.220	1.059
Singapore	1.108	0.987	1.089	1.030
Thailand	1.129	0.984	1.132	1.013
Viet Nam	1.494	1.149	1.249	1.041

**Table 4:** E-waste intensity comparison among ASEAN and selected high-income countries.

Country		E-Waste Intensity (Thousand Ton/Billion US Dollar)	
		2015	2019
ASEAN	Brunei Darussalam	0.587	0.643
	Cambodia	0.817	0.908
	Indonesia	1.411	1.343
	Lao PDR	0.964	1.288
	Malaysia	0.908	0.912
	Myanmar	0.768	0.943
	The Philippines	1.214	1.178
	Singapore	0.341	0.337
	Thailand	1.394	1.372
	Viet Nam	1.113	1.280
(Selected) High-income countries	Republic of Korea	0.527	0.552
	Japan	0.423	0.414
	The United Kingdom	0.542	0.547
	France	0.465	0.458
	United States	0.389	0.378

## Data Availability

Publicly available datasets were analyzed in this study. This data can be found here: https://globalewaste.org (E-waste data by country; accessed on 3 August 2021) and https://data.worldbank.org (GDP and population data by country; accessed on 3 August 2021).

## Appendix A

The results of additive LMDI decomposition analysis from 2015 (base year) to each comparison year are shown in Figure A1. Since Brunei Darussalam had the same amount of E-waste generated in 2015 and 2016, Figure A1a only shows the analysis result starting from the decomposition between 2015 and 2017.
